# Can Teacher Support Alleviate Academic Anxiety in Chinese as a Foreign Language Learners? The Chain Mediating Role of Self-Efficacy and Academic Emotions

**DOI:** 10.3390/bs16040565

**Published:** 2026-04-09

**Authors:** Xinying Lyu, Xiaojun Yin, Yuchen Yang

**Affiliations:** 1 College of International Education, China University of Petroleum-Beijing, Beijing 102249, China; xylyu@cup.edu.cn; 2 College of International Chinese Studies, Beijing Language and Culture University, Beijing 100083, China; 202521100104@stu.blcu.edu.cn

**Keywords:** academic anxiety, teacher support, self-efficacy, academic emotions

## Abstract

Academic anxiety is a negative emotional state experienced by learners of Chinese as a foreign language (CFL) during their Chinese language learning process. To explore the mediating mechanisms of positive and negative emotions and academic self-efficacy between teacher support and academic anxiety, a questionnaire survey was conducted among 1047 CFL learners, and a structural equation model was established to test the mediating effects. The study found that teacher support helps alleviate academic anxiety among CFL learners; teacher support exerts a certain inhibitory effect on learners’ academic anxiety. However, teacher support does not directly reduce academic anxiety but indirectly influences it through the independent mediating roles of self-efficacy and academic emotions, as well as through the chain mediating effect of both. Based on these findings, the paper provides recommendations for maintaining positive emotions, enhancing self-efficacy, implementing tailored teacher support, and constructing comprehensive motivational mechanisms for CFL learners.

## 1. Introduction

Anxiety is a complex and unpleasant emotional state, often arising when individuals feel powerless in the face of challenges, fail to meet their anticipated goals, or perceive a threat to something they value (Jiang & Wu, 2017). As a specific type of anxiety, academic anxiety in language learning manifests as tension, fear, and dread, closely linked to an individual’s confidence in their language abilities, fear of failure, and adaptation to the language use environment. This form of anxiety is related to learners’ self-confidence, self-efficacy, and past language learning experiences. High levels of anxiety may lead learners to avoid using the foreign language, consequently hindering the development of language skills. Both input (e.g., reading) and output processes (e.g., speaking) in foreign language acquisition can trigger academic anxiety (Dewaele, 2019). In recent years, the broader impact of anxiety within Chinese as a second language acquisition has increasingly gained scholarly attention (Qiao et al., 2025); however, studies focusing specifically on the underlying mechanisms of their academic anxiety remain scarce.

Teacher support, a crucial source of social support in the learning process, includes caring, listening, understanding, nurturing, and encouragement. Learners perceive it as the supportive actions provided by teachers during their educational journey (Y. R. Li et al., 2023). Teacher support is a multidimensional construct that can be broadly categorized into three core types: emotional, academic, and structural support (Martin & Rimm-Kaufman, 2015; Q. Zhang & Wang, 2023). Specifically, emotional support focuses on fostering a connection between teachers and learners, enhancing learners’ sense of belonging and security. Academic support emphasizes providing clear academic guidance and feedback, playing a crucial role in boosting learners’ self-efficacy to address academic challenges. Structural support involves establishing clear learning rules, goals, and expectations to help learners plan their tasks more effectively. Current research indicates that integrating these dimensions of support significantly helps learners overcome academic obstacles and improves their overall academic performance (Q. Zhang & Wang, 2023). According to self-determination theory, teacher support plays a key role in satisfying the basic psychological needs of learners, thereby fostering positive and autonomous learning motivation. The multiple dimensions of social support, including teacher support, significantly positively influence learning motivation (Deci, 2004). For CFL learners, effective alleviation of academic anxiety relies on both internal guidance and external support. The current academic discourse on communicative mitigation of academic anxiety is vague, with perspectives yet to be empirically validated, making it challenging to unveil the deeper mechanisms at play. Given this context, this study utilizes survey data and a structural equation model (SEM) to test theoretical hypotheses and investigate the impact of teacher support on the academic anxiety of CFL learners.

## 2. Literature Review and Research Questions

### 2.1. Academic Anxiety

Academic anxiety is a negative emotional state experienced by students in academic activities (Pekrun, 2006). Scholars suggest that effective communication involves complex, non-instantaneous mental operations. Using a second language may trigger self-assessment of communicative competencies, resulting in silence, heightened self-awareness, fear, and panic, thus leading to “foreign language anxiety”. Recent neurocognitive research has deepened our understanding of this mechanism; an ERP study on Chinese word processing revealed that learners with high trait anxiety exhibit a specific “vigilance-avoidance” neural pattern, consuming excessive cognitive resources during the early stages of processing emotion-laden words, which impedes subsequent deep semantic integration (J. Liu & Fan, 2026). Furthermore, the modern academic environment introduces new stressors; Al-Osail et al. (2026) identified that “phubbing” (phone snubbing) behavior in educational settings significantly fuels foreign language anxiety by disrupting social connectedness and exacerbating academic delay behaviors.

Emotions involve a series of subjective feelings that engage cognitive, physiological, and affective aspects. Studies have shown that different emotional experiences during the learning process affect learners’ motivation and thus their level of engagement (Pekrun et al., 2002). Furthermore, specific negative emotions, such as shame and anxiety, may paradoxically enhance motivation and prompt learners to adjust their behaviors (X. Wang et al., 2023), suggesting that negative emotions can play a constructive role in certain contexts by triggering reflection and changes in learning approaches (Z. S. Zhang & Li, 2018). However, neural evidence suggests that anxiety can disrupt high-level cognitive functions required for bilingual tasks; Zhong et al. (2026) demonstrated that the neural costs of code-switching are significantly modulated by the speaker’s emotional state and processing efficiency.

Foreign language anxiety, as a subset of academic emotions, comprises persistent emotional responses such as tension, unease, and worry, primarily triggered by challenges in the learning process, unsatisfactory academic performance, or negative external evaluations (Meng et al., 2023). Recently, academic anxiety has become a focal issue in second language acquisition research. Thus, academic anxiety is a negative emotional reaction arising from learners’ perceptions of their academic environment, activities, achievements, and external evaluations.

### 2.2. Teacher Support

Teacher support refers to actions perceived by learners as supportive, offered by teachers in academic or personal contexts (Leflot et al., 2011). It represents a primary source of support outside of the family. Recent empirical evidence highlights the nuance in how support is delivered; utilizing latent profile analysis, Zhou et al. (2025) found that students perceiving a “balanced” profile of teacher support (integrating both instrumental and emotional dimensions) exhibited significantly lower anxiety and higher academic engagement compared to other profiles.

The role of teacher support in promoting engagement has been widely acknowledged in academia. As a key variable, teacher support significantly impacts the learners’ process, closely associated with their emotional attitudes. Positive teacher support correlates significantly with positive academic emotions (such as interest, hope, satisfaction, enjoyment, and self-esteem) and inversely with negative academic emotions (including anxiety, depression, inferiority, and anger) (Sun, 2023). Moreover, longitudinal research by Ye et al. (2025) indicates that the protective role of teacher support extends over time, fostering “academic buoyancy”—the capacity to bounce back from setbacks—which mediates the relationship between self-efficacy and the reduction in academic burnout. Indeed, recent network analyses utilizing the Control-Value framework reveal that negative states like burnout often form a “hidden architecture” that deeply compromises university students’ psychological functioning, further highlighting the necessity of robust environmental support (Demeter et al., 2026). In educational settings, teachers’ care and guidance in facing challenges play a crucial role in helping learners develop resilience and openness in their personalities (Bao, 2023). High-quality teacher-student relationships, especially those built on teacher responsibility and learners’ affection for teachers, significantly affect learners’ engagement and willingness to learn (Yang et al., 2020).

### 2.3. Academic Emotions

Academic emotions are those directly related to academic activities and outcomes (Pekrun, 2006). Fredrickson’s (2001) broaden-and-build theory and Pekrun’s (2006) control-value theory are widely recognized as core frameworks in emotion research (MacIntyre & Gregersen, 2012; Shao et al., 2019). Fredrickson’s (2001) broaden-and-build theory highlights the role of positive emotions, like pleasure, in expanding cognitive and action resources, thereby fostering psychological resilience and resource development. Conversely, negative emotions, like anxiety, may lead to a contraction of resources. Within this framework, MacIntyre and Gregersen (2012) observed that language learners often experience both positive and negative emotions, revealing the complexity and multidimensionality of emotions in language learning. Expanding on this, recent scholarship has highlighted “epistemic emotions” such as curiosity and boredom; Nikitina et al. (2025) found that curiosity acts as a vital counter-force to boredom, sustaining motivation even in challenging L2 learning contexts. The control-value theory defines academic emotions across three core dimensions: the arousal object (i.e., the specific stimulus or situation, such as a language task, that triggers the emotional response) oriented towards a specific goal, the valence (positive or negative) of the emotion, and the intensity or arousal level of the emotion (Pekrun, 2006). Within this framework, pleasure is seen as a positive, affect-focused process emotion, while anxiety is viewed as a negative, outcome-related emotion. From a cognitive semantic perspective, the very conceptualization of these emotions is culturally constructed; Kacprzak (2025) illustrates how emotion concepts (e.g., Emocja) undergo semantic shifts driven by cultural changes, suggesting that a learner’s internal definition of “anxiety” or “joy” is deeply rooted in their linguistic and cultural background. These theories underscore the significant impact of both positive and negative emotions on individual short-term and long-term cognitive and action resources and learning outcomes, highlighting the pivotal role of emotions in language acquisition. Employing Fredrickson’s broaden-and-build theory and Pekrun’s control-value theory as the theoretical framework, this study aims to explore how foreign language pleasure and anxiety influence learners’ perception of teacher support. This approach allows for an in-depth analysis of the mechanisms by which emotions influence learners’ willingness to communicate in Chinese, particularly in understanding how emotions affect cognitive and action resources and shape communicative behaviors in language learning. On this basis, the study seeks to reveal the specific role of learners’ emotional experiences in Chinese learning and their impact on communicative willingness, thereby offering insights into the management of emotions in Chinese language learning and teaching practices.

### 2.4. Self-Efficacy

In the context of the development of positive psychology, the relationship between academic anxiety and self-efficacy (Bandura et al., 2001) has been debated. It serves as the domain-specific manifestation of learners’ general confidence within academic settings (Xiao & Liu, 2017). In the era of digital education, this construct has evolved; Q. Zhang et al. (2025) introduced the concept of “AI learning self-efficacy,” demonstrating that high AI literacy reduces classroom anxiety and enhances willingness to communicate, specifically by boosting learners’ confidence in navigating technological tools.

The formation and development of self-efficacy are influenced by the outcomes of individual learning behaviors, with academic performance significantly affecting self-efficacy (H. Li, 2016). Self-efficacy directly impacts learners’ effort, perseverance in overcoming difficulties, and emotions (calmness or anxiety) during learning, and is a key factor in enhancing learning motivation and performance (Guo et al., 2023). For instance, learners with high self-efficacy tend to invest more time in learning (Gong et al., 2023), adopt more suitable and effective autonomous learning strategies (Cui & Hu, 2022; H. Wang et al., 2021), and exhibit lower levels of anxiety (Yuan & Wang, 2022), leading to higher academic achievements.

### 2.5. Academic Anxiety and Teacher Support

The interplay between teacher behavior and learner emotions is complex. Academic emotions, categorized by valence and arousal levels, fall into four types: positive-low arousal, negative-low arousal, positive-high arousal, and negative-high arousal. Academic anxiety is classified as a negative-high arousal emotion (Ma, 2021). To illustrate the other quadrants, enjoyment represents positive-high arousal, relaxation is positive-low arousal, and boredom serves as a typical negative-low arousal emotion. Learners’ emotional states reflect their preferences towards instructional content, media, and environments, assisting educational agents in understanding cognitive styles and learning interests. Additionally, analyzing emotional states reveals how educational environments impact learner emotions, subsequently influencing their motivation (H. X. Liu et al., 2023). High academic anxiety often arises when learners place great importance on current tasks but feel a lack of control, potentially adversely affecting their communicative willingness. Academic emotions, by modulating cognitive and motivational mechanisms, impact learners’ processes and achievements. Academic anxiety, specifically, can lead to attentional diversion from learning tasks, impaired self-management, and decreased communicative willingness, ultimately affecting academic achievement. According to social support theory, support behaviors perceived or received in social relationships, such as teacher and peer support, promote psychological health and development. Empirical studies have found that teacher support can lower learners’ anxiety levels and mitigate the adverse psychological impact of negative events (R. Zhang et al., 2020). Based on these findings, the following question is proposed:

**H1.**  *Teacher support has a significant negative impact on academic anxiety among CFL learners.*

### 2.6. Teacher Support, Self-Efficacy, and Academic Anxiety

Self-efficacy refers to an individual’s belief in their academic abilities to organize and execute the courses of action required to complete learning tasks (Bandura et al., 2001; Bian, 2004). It represents a subjective evaluation of one’s capability to control learning behavior and outcomes. In the context of the development of positive psychology, the relationship between academic anxiety and self-efficacy has been debated. As a vital psychological resource, self-efficacy, highlighted in the Health Belief Model and Protection Motivation Theory, is deemed to exert influence on mental health. Studies indicate that individuals with higher self-efficacy possess better emotional regulation and resilience, enabling them to mobilize more resources against adversity, thus alleviating anxiety (Chen, 2019; Yao & Ma, 2021). Generally, self-efficacy significantly negatively impacts individuals’ anxiety.

According to Bandura’s theory, self-efficacy beliefs arise from a complex self-persuasion process involving cognitive processing of various functional information from positive social interactions and physiological feedback (Bandura, 1993). Once established, these beliefs significantly enhance functional levels and quality. In this context, teachers’ trust, care, positive evaluations, and feedback have been shown to effectively boost learners’ academic self-efficacy (Alivernini & Lucidi, 2011). Teacher support in school and classroom environments is a key factor affecting learners’ self-efficacy, significantly positively impacting their academic self-efficacy (Ji & Zhao, 2021). Accordingly, the study hypothesizes:

**H2.**  *Teacher support significantly positively influences self-efficacy.*

**H3.**  *Self-efficacy significantly negatively impacts academic anxiety among CFL learners.*

### 2.7. Teacher Support, Academic Emotions, and Academic Anxiety

Academic emotions in learning contexts, encompassing learning, teaching, and achievement aspects, include both positive and negative emotions. Studies show that positive academic emotions enhance motivation, learning effectiveness, and performance (R. D. Liu et al., 2018; S. J. Zhang et al., 2016), while negative emotions may adversely impact performance (Lichtenfeld et al., 2012) and intensify academic anxiety. Regarding the relationship between teacher support and academic emotions, research on cognitive-behavior therapy suggests that maladaptive cognitive patterns often underlie adverse behaviors and emotions. Through engaging in communicative activities and using social media in the target language country (G. X. Zhang & Xin, 2022), individuals can mimic and learn new role behaviors, internalizing positive cognitive patterns and thus significantly improving emotional wellbeing. Learners engaged in communicative language tasks facing academic setbacks are likely to mobilize positive academic emotions. Consequently, the study proposes the following hypotheses:

**H4.**  *Teacher support significantly positively influences positive academic emotions and negatively influences negative academic emotions.*

**H5.**  *Positive academic emotions significantly negatively impact academic anxiety among CFL learners, whereas negative academic emotions significantly positively impact it.*

### 2.8. Teacher Support, Self-Efficacy, Academic Emotions, and Academic Anxiety

Previous research has identified a causal relationship between self-efficacy and academic emotions, but the direction of influence varies across different academic emotions. Studies suggest that individuals with high self-efficacy, when facing stressful events, are more confident in handling adversities, viewing problems as challenges, whereas those with low self-efficacy tend to perceive the same events as threats or uncontrollable situations. Thus, individuals with high self-efficacy believe in their problem-solving abilities, exhibiting more positive emotional attitudes; conversely, those with low self-efficacy fall into self-doubt, feeling incapable of handling challenges, leading to negative coping strategies like avoidance or withdrawal (Sutherland et al., 2023). According to the stress appraisal theory, the perception of stressors is influenced by self-efficacy. Studies show that higher self-efficacy can moderate the relationship between stressors and stress responses, making individuals less prone to tension and anxiety (Peng et al., 2015). Individuals with high self-efficacy tend to view stressors as manageable challenges, while those with low self-efficacy may feel powerless, perceiving them as insurmountable obstacles, leading to significant stress responses. This finding underscores the critical role of self-efficacy in coping with stress and anxiety. Based on these considerations, the study proposes the following hypotheses:

**H6.**  *Self-efficacy significantly positively influences positive academic emotions and negatively influences negative academic emotions.*

**H7.**  *Self-efficacy and academic emotions play a sequential mediating role in the impact of teacher support on academic anxiety among CFL learners.*

In summary, the literature reviewed above conceptually informs the development of the proposed theoretical model. While previous studies have primarily offered descriptive accounts of individual variables, this research analytically integrates them to construct a chain mediation framework. Specifically, environmental factors (teacher support) do not operate in isolation; rather, they serve as distal antecedents that must be cognitively processed through the learner’s self-appraisal (self-efficacy) to effectively regulate subsequent emotional states (academic emotions), which ultimately predict the alleviation of academic anxiety.

**Figure 1:**
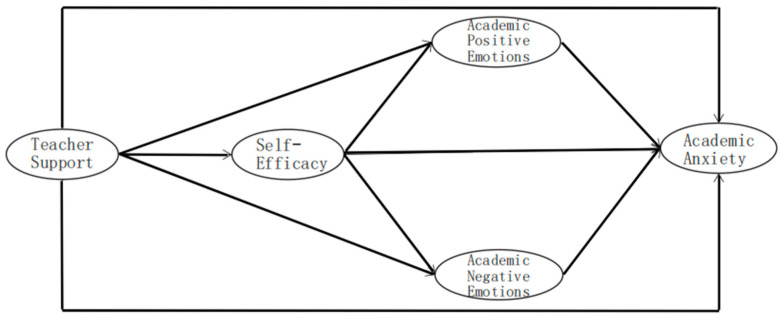
Theoretical Model Diagram.

Based on the aforementioned hypotheses, this study constructs the theoretical model depicted in Figure 1.

## 3. Method

### 3.1. Participants

This study employed convenience sampling to collect 1101 questionnaires. Following the principles outlined in previous research, after eliminating responses with overly short completion times and invalid questionnaires, a total of 1047 valid responses were obtained. For detailed information about the survey participants, see Table 1.

### 3.2. Instruments

The international Chinese language learners participating in the questionnaire had all passed HSK Level 4, demonstrating a certain level of Chinese language proficiency. The questionnaire was provided in both Chinese and English and was distributed via the “Wenjuanxing” platform, ensuring that the participants could accurately comprehend it and respond according to their language preference. The scales used were primarily translated and adapted from well-established domestic and international scales. The instrument included four measured variables and employed a five-point Likert scale, with responses ranging from “1” (strongly disagree) to “5” (strongly agree).

The Academic Anxiety Scale was adapted from the Adolescent Academic Emotion Questionnaire developed by Y. Dong and Yu (2010), which included seven items related to academic anxiety, such as “I feel anxious when my grades decline,” with a reliability coefficient of 0.76. The Teacher Support Scale was based on the scale developed by Chai and Gong (2013), modified to suit the characteristics of international Chinese learners, and consisted of seven items addressing autonomy support, cognitive support, and emotional support, such as “My teacher provides timely feedback on learning tasks or assignments,” with a reliability coefficient of 0.72. The Self-Efficacy Scale was adapted from the Adult Self-Efficacy Scale developed by J. Li et al. (2015), with adjustments made based on interview data and specific teaching contexts. This scale measured self-efficacy in two dimensions: learning ability self-efficacy and academic self-efficacy, comprising 12 items, such as “I believe I can analyze long-term problems and find solutions,” with a reliability coefficient of 0.69. The Academic Emotion Questionnaire (AEQ), developed by Pekrun et al. (2009), was used to measure academic emotions in two dimensions: positive and negative academic emotions, with a total of 12 items.

### 3.3. Methodology

The study utilized Stata 14 software for multilevel linear regression analysis, employing a hierarchical linear model (HLM) to test the theoretical model. In this model, self-efficacy and achievement goal orientation were treated as between-person variables, while academic anxiety and learning motivation were considered within-person, repeatedly measured variables. Given the multilevel nature of the sample data, within-person variables were nested within between-person variables. Independent and dependent variables were at the first level (within-person), while moderating variables were at the second level (between-person). To mitigate potential multicollinearity effects, academic anxiety was group-mean centered, while self-efficacy and achievement goal orientation were grand-mean centered. Interaction terms of these variables were then calculated. Prior to testing the multilevel model, a null model of learning motivation was analyzed to estimate the proportion of within-person and between-person variance.

For further in-depth analysis, preliminary data processing was conducted using SPSS 27.0. The study employed a comprehensive application of Hayes’ SPSS macro PROCESS and Amos 26.0 software. To ensure methodological transparency and reproducibility, the analytical procedures were strictly standardized. Missing data were handled using Maximum Likelihood (ML) estimation. Furthermore, the evaluation of the structural equation model’s fit relied on established indices, including the Comparative Fit Index (CFI), Tucker–Lewis Index (TLI), and Root Mean Square Error of Approximation (RMSEA). The constructed questions model, integrating survey and behavioral data results, systematically analyzed the factors influencing learning motivation and academic achievement. The reliability and validity of measurement tools were verified through Cronbach’s alpha coefficients and confirmatory factor analysis. Quantification of self-efficacy and academic emotions was based on the mean scores of respective scales.

## 4. Research Findings

### 4.1. Descriptive Statistics and Correlation Analysis Among Variables

Table 2 presents the descriptive statistics and correlation analysis results for variables including gender, age, grade level, subject and major, teacher support, self-efficacy, positive academic emotions, negative academic emotions, and academic anxiety. In terms of mean values, gender (*M* = 3.117, *SD* = 0.478) and age (*M* = 3.094, *SD* = 0.684) are close to the median, indicating a relatively balanced distribution of gender and age within the sample. The mean for grade level (*M* = 2.767, *SD* = 0.677) is slightly below the median, suggesting that the grade levels in the sample are somewhat concentrated.

Teacher support (*M* = 3.845, *SD* = 0.651) shows a relatively high mean, indicating that most students perceive strong support from their teachers. Similarly, self-efficacy (*M* = 3.514, *SD* = 0.684) is also relatively high, suggesting that students generally feel confident in their academic abilities. The mean for positive academic emotions is 2.214 (*SD* = 0.768), slightly below the median, indicating that students experience relatively few positive emotions in their academic pursuits. Negative academic emotions (*M* = 2.109, *SD* = 0.748) and academic anxiety (*M* = 2.014, *SD* = 0.814) show lower means, suggesting that students in the sample generally experience lower levels of negative emotions and anxiety.

The correlation analysis shows a significant positive correlation between teacher support and both self-efficacy and positive academic emotions (*r* = 0.402 **, *r* = 0.467 **), indicating that teacher support has a beneficial impact on students’ confidence and emotional state. In contrast, the correlations between teacher support and negative academic emotions and academic anxiety are relatively weak (*r* = −0.015 **, *r* = −0.019 **), suggesting that teacher support has a limited direct effect on negative emotions. Additionally, the correlations between background variables such as gender and age and academic emotions or anxiety are also low, indicating that these background variables have a relatively minor impact on emotions and anxiety.

These findings highlight the important role of teacher support in enhancing students’ self-efficacy and positive emotions. Further analysis using structural equation modeling could explore the specific pathways through which teacher support, self-efficacy, and emotional variables interact.

It is observed that academic anxiety shows a significant negative correlation with age (−0.077 **) and grade level (−0.081 **), indicating that older CFL learners and those with higher educational levels typically experience lower levels of academic anxiety. This may be attributed to the increased maturity and experience in handling academic pressures and challenges that come with age, and the stronger learning skills and coping strategies associated with higher educational levels, thereby reducing anxiety. Additionally, a positive correlation between self-efficacy and gender (0.271 **) and grade level (0.104 **) reveals the positive impact of gender and educational level on learners’ self-efficacy. While initial observations suggest that demographic variables, such as gender and chosen field of study, may introduce natural variance in learners’ academic self-efficacy, identifying specific subgroup disparities falls outside the primary scope of the current structural equation modeling. Rather than focusing on demographic comparisons, the present study strictly aims to elucidate the universal, cross-demographic psychological mechanism—specifically, how the chain mediation of self-efficacy and academic emotions operates within the overarching Control-Value Theory framework. A strong positive correlation between positive academic emotions and teacher support (0.467 **) suggests that learners more interested in Chinese communication tend to have more positive academic attitudes, likely due to increased engagement and satisfaction with the learning content. Furthermore, a moderate positive correlation between negative academic emotions and academic anxiety (0.141 **) implies that these may exacerbate each other, suggesting that negative emotions can lead to an increase in academic anxiety, and vice versa.

### 4.2. Structural Equation Model Analysis

**Figure 2:**
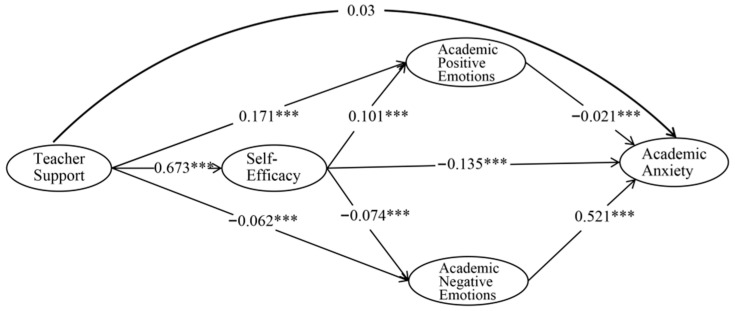
Structural Equation Model of Teacher Support on Academic Anxiety among CFL Learners. *** *p* < 0.001.

By constructing the structural equation model shown in Figure 2, and controlling for demographic variables such as gender, age, education level, and field of study, the model fit indices demonstrated a good fit. First, the analysis revealed that teacher support did not directly predict academic anxiety among international Chinese language learners (*β* = 0.03, *p* > 0.05), indicating no direct causal relationship between the two, and thus, Hypothesis H1 was not supported. However, the total effect analysis showed that teacher support had a significant negative impact on academic anxiety, suggesting a negative causal relationship between teacher support and academic anxiety.

Second, teacher support significantly positively predicted self-efficacy (*β* = 0.673, *p* < 0.001), and self-efficacy significantly negatively predicted academic anxiety (*β* = −0.135, *p* < 0.001), thus supporting Hypotheses H2 and H3. Third, teacher support positively predicted positive academic emotions (*β* = 0.171, *p* < 0.001) and negatively predicted negative academic emotions (*β* = −0.062, *p* < 0.001). Positive academic emotions significantly negatively predicted academic anxiety (*β* = −0.021, *p* < 0.001), while negative academic emotions positively predicted academic anxiety (*β* = 0.521, *p* < 0.001), supporting Hypotheses H4 and H5.

Finally, self-efficacy significantly positively predicted positive academic emotions (*β* = 0.101, *p* < 0.001) and negatively predicted negative academic emotions (*β* = −0.074, *p* < 0.001), supporting Hypothesis H6. Overall, the model demonstrates that self-efficacy and academic emotions play significant mediating roles in the relationship between teacher support and academic anxiety.

Table 3 tested the mediating effects of teacher support, self-efficacy, and academic emotions in the relationship between teacher support and academic anxiety using the bias-corrected percentile Bootstrap method (with 5000 resamples). The results showed that all indirect effect pathways were significant. Teacher support indirectly influenced academic anxiety through positive academic emotions, accounting for 2.17% of the total effect, with a 95% confidence interval of [−0.004, −0.002], *p* < 0.05. The chain mediation effect of teacher support through self-efficacy and positive academic emotions accounted for 1.71% of the total effect, with a 95% confidence interval of [−0.005, −0.002], *p* < 0.001.

Teacher support directly and negatively predicted academic anxiety through self-efficacy, with an indirect effect accounting for 47.85% of the total effect, making this the most substantial pathway, with a 95% confidence interval of [−0.041, −0.037], *p* < 0.05. The indirect effect of teacher support on academic anxiety through negative academic emotions accounted for 19.01% of the total effect, with a 95% confidence interval of [−0.022, −0.019], *p* < 0.05. Finally, the chain mediation effect of teacher support through self-efficacy and negative academic emotions accounted for 29.26% of the total effect, with a 95% confidence interval of [−0.034, −0.031], *p* < 0.05.

These results suggest that self-efficacy plays a critical mediating role in the relationship between teacher support and academic anxiety, significantly influencing academic anxiety levels by modulating both positive and negative academic emotions.

## 5. Discussion

### 5.1. The Distal Nature of Teacher Support and the Validity of Full Mediation

A pivotal finding of this study is that perceived teacher support does not exert a significant direct effect on academic anxiety (*β* = 0.03, *p* > 0.05), but rather influences it exclusively through a full mediation mechanism involving self-efficacy and academic emotions. This result offers empirical validation for the environmental postulates of the Control-Value Theory (CVT) within the specific context of CFL. According to CVT, environmental factors such as teacher support function as “distal antecedents” rather than proximal determinants of achievement emotions (C. Li, 2025; R. Wang & Xu, 2026). While teachers provide essential scaffolding and emotional resources, these external inputs must be cognitively processed by the learner to alter their “control appraisal” (perceived competence) before they can effectively mitigate anxiety (B. J. Xu, 2024).

The absence of a direct effect suggests that teacher support, in isolation, does not mechanically reduce the psychological burden of learning Chinese. Instead, its efficacy is contingent upon its ability to be internalized by the learner. If teacher support fails to translate into an enhanced sense of control (i.e., self-efficacy), the “threat” appraisal associated with the high cognitive load of CFL learning remains active, sustaining high levels of anxiety. This interpretation aligns with B. J. Xu’s (2024) finding that teacher support impacts academic achievement solely by regulating learners’ psychological states, reinforcing the view that external support must be catalyzed into internal agency to be effective.

### 5.2. Self-Efficacy as a Cognitive Shield in CFL Acquisition

The structural model identifies self-efficacy as the dominant mediator, accounting for the largest proportion of the indirect effect (47.85%). This underscores the critical function of self-efficacy as a “cognitive shield” against Foreign Language Anxiety (FLA). In the acquisition of Chinese, where learners face the unique “zero-transfer” challenge of the logographic writing system and tonal phonology, the perception of task difficulty often creates a high-anxiety environment. Consistent with the meta-analytic findings of Qiao et al. (2025), which identify beginners as the demographic most vulnerable to anxiety, our results suggest that self-efficacy counteracts this vulnerability by enhancing the learner’s control appraisal. When learners believe in their capability to master specific linguistic tasks, the “threat value” of the language is diminished, directly inhibiting the generation of anxiety. Conversely, without this boost in self-efficacy, even high levels of external support may fail to alleviate the learner’s apprehension regarding failure.

### 5.3. The Chain Mediation Mechanism of Academic Emotions

This study further elucidates the complex internal process through the chain mediation pathway: Teacher Support → Self-Efficacy → Academic Emotions → Academic Anxiety. This pathway demonstrates that self-efficacy not only directly suppresses anxiety but also fosters a positive emotional landscape that serves as a buffer. Specifically, teacher support enhances self-efficacy, which triggers positive academic emotions (e.g., enjoyment); these positive emotions, in turn, reduce anxiety. This supports the “Broaden-and-Build” perspective within the CVT framework, as observed in recent chain mediation models (Song & Wang, 2026). Positive emotions generated by high efficacy function to broaden the learner’s cognitive resources, allowing for greater resilience against the stressors of language learning. This finding highlights that the reduction in anxiety is not limited to removing negative stimuli but involves the active cultivation of positive psychological resources (efficacy and enjoyment) facilitated by the teacher.

## 6. Conclusions and Implications

### 6.1. Conclusions

This study empirically investigated the mechanisms through which teacher support alleviates academic anxiety in CFL learners. First, while the structural model revealed no significant direct path from teacher support to academic anxiety (leading to the rejection of H1), the total effect was significantly negative. This indicates that teacher support acts as a crucial foundational resource that exerts a robust indirect inhibitory effect on anxiety. This finding effectively contextualizes and extends previous assertions that supportive interpersonal environments—characterized by assistance, recognition, and positive evaluation—are critical for affective regulation and meaningful language output (Wentzel et al., 2016).

Second, the structural equation model highlights self-efficacy as the primary independent mediator. Teacher support was found to significantly enhance learners’ self-efficacy (supporting H2), which in turn substantially mitigated academic anxiety (supporting H3). This pathway accounted for the largest proportion of the indirect effect, corroborating recent studies which emphasize that external pedagogical support must be internalized as academic confidence to effectively buffer against learning stress (X. Dong & Zhang, 2023; Fu & Qi, 2023; Xie et al., 2023).

Third, the study clarifies the complex affective pathways involved, fully mapping the scope of the remaining hypotheses. Teacher support significantly fostered positive academic emotions(supporting H4), both of which directly predicted lower academic anxiety (supporting H5). Furthermore, a robust chain mediating mechanism was validated (supporting H7): enhanced self-efficacy not only directly reduced anxiety but also triggered positive emotions and suppressed negative ones (supporting H6). This suppression is particularly vital given that recent scholarship has increasingly highlighted the detrimental effects of other under-researched negative academic emotions, such as boredom, on student engagement in second language acquisition (Kruk et al., 2021; Pawlak et al., 2020; Zawodniak & Kruk, 2020). Given that foreign language learning often inherently provokes tension and communication apprehension due to lower proficiency and cultural barriers (Dewaele, 2019; Elkhafaifi, 2005), these comprehensive findings confirm that external support is most effective when it synergistically activates the learner’s cognitive (self-efficacy) and emotional resources.

### 6.2. Implications

Theoretically, these findings significantly enrich the understanding of cognitive and emotional processes in second language acquisition. By elucidating the chain mediation mechanism, this study demonstrates that affective regulation within learning contexts is not merely a direct response to external stimuli (teacher support). Instead, it is a highly cognitive process that is contingent upon the activation and enhancement of the learner’s internal self-efficacy, offering robust empirical support for the cognitive-motivational theories of academic emotions.

Facing challenges in learning, the emotional states and self-efficacy of CFL learners are particularly crucial. Maintaining positive emotions and enhancing self-efficacy help these learners to more effectively address problems in the learning process. Incorporating knowledge from positive psychology, reading or watching motivational stories and videos, and strengthening self-motivation and emotional regulation capabilities are vital for better handling learning difficulties and challenges. Learners need to continually improve their self-efficacy, maintain a proactive attitude towards learning challenges, and bolster their confidence in ongoing learning through problem-solving. The mediating role of academic self-efficacy indicates a sequential mechanism: teacher support effectively enhances learners’ academic self-efficacy, which in turn directly reduces their academic anxiety levels. Previous studies consider academic self-efficacy as an intermediary variable in the relationship between perceived interpersonal environment and positive outcomes like academic performance (Z. Z. Xu & Qi, 2019). This implies that learners’ perceptions and psychological responses to interpersonal interactions within educational environments, bridged by self-efficacy, significantly impact their academic achievements. Self-efficacy not only determines learners’ interpretations and responses to educational experiences but is also a key factor affecting their academic performance. Therefore, enhancing learners’ academic self-efficacy is not only a direct path to improving academic performance but also a crucial strategy for reshaping their cognition and engagement in learning environments. Additionally, summarizing and accumulating problem-solving experiences, enhancing learning capabilities, and engaging in communicative exchanges to build supportive learning networks are key to enhancing learning motivation and engagement.

This study further highlights the critical role of teacher support in regulating learners’ academic emotions and anxiety. Given that teacher support is a multidimensional construct, educational practice should adapt to individual learner needs. For instance, when learners face significant psychological stress, providing targeted emotional support can effectively alleviate their anxiety. In contrast, when dealing with specific language barriers, explicit academic support and structural support (e.g., clear learning goals and feedback) can enhance learners’ self-efficacy and performance. Therefore, CFL teachers must apply these different dimensions of support flexibly to achieve optimal educational outcomes.

Emphasizing the development of CFL teachers’ problem awareness, research methods, reflective abilities, and digital teaching competencies is essential, as well as enhancing their environmental adaptability, cross-cultural communication abilities, and continuous development capabilities (H. Wang, 2023). Teachers play a vital role in learners’ Chinese learning journey, recognizing the malleability of language learning engagement (Mystkowska-Wiertelak, 2020). By providing cognitive response regulation strategies, teachers can positively influence learners’ sense of ability, control, and effort, thereby positively affecting their self-efficacy, and implementing customized teaching support (Shi & Cui, 2023). Teachers should focus on learners’ academic emotions, especially giving special attention to those exhibiting academic anxiety. Academic anxiety may stem from unclear learning goals, low-challenge tasks, insufficient support, and inadequate expectations of success. Self-efficacy, as a belief in one’s own ability to succeed, is closely related to achievement motivation—the intrinsic drive to pursue success. Hence, the teacher’s role is not only to provide external support but, more importantly, to stimulate learners’ intrinsic learning motivation. To achieve this, teachers need to create a learning motivation mechanism that integrates internal and external factors, including setting clear learning objectives, providing challenging tasks, timely feedback, emphasizing the importance of learning, and recognizing and praising learners’ achievements. Such mechanisms can effectively alleviate learners’ academic anxiety, enhance their perception of teacher support, and consequently promote academic achievement.

### 6.3. Research Limitations and Future Prospects

However, several methodological limitations must be explicitly acknowledged. First, the cross-sectional design of this study restricts the ability to draw definitive causal inferences regarding the relationships among teacher support, self-efficacy, academic emotions, and academic anxiety. Second, the reliance on self-report measures may introduce common method bias and subjective reporting inaccuracies. Future longitudinal or experimental studies utilizing multi-informant approaches are required to rigorously validate the causal pathways proposed in this structural model.

Considering the impact of teaching factors such as teaching modes and platform functionalities on learners’ academic anxiety, motivation to speak, and their interrelationships, the findings of this study need to be validated and expanded in a broader range of teaching contexts. Additionally, while the study focuses on the relationship between teacher support and academic anxiety, it does not fully explore other external and internal factors beyond teacher support that might affect these elements, such as peer influence, school environment, specific teaching methods, or individual learner differences. Furthermore, the current study did not explicitly measure participants’ specific native languages; given the highly multilingual backgrounds of many international CFL learners, future research should incorporate detailed linguistic profiling to better control for potential cross-linguistic influences. Therefore, for a more comprehensive understanding, future research should more thoroughly examine these factors and may consider expanding the sample range to enhance the generalizability and applicability of the findings.

The study results theoretically corroborate the control-value theory of academic emotions and the cognitive-motivational model, offering a new perspective for the localization of academic emotion theory. Furthermore, these findings have implications for CFL education practice. Firstly, employing diversified assessment methods to evaluate learners’ academic achievements is crucial, with a need to encourage learners more, allowing them to experience success and positive emotions. In this way, positive academic emotions can strengthen intrinsic motivation, prompting learners to adopt higher-order learning strategies, which not only facilitate cognitive activities but also foster proactive learning attitudes. On the other hand, when learners perform poorly in exams, improving their learning motivation and strategies can effectively reduce the academic anxiety brought about by exam failure. Additionally, intervening in learners’ negative emotions and cultivating their emotional regulation abilities can inspire their passion for learning and improve learning methods.

Furthermore, while our structural model posits that self-efficacy predicts academic positive and negative emotions based on the Control-Value Theory, we acknowledge the theoretical possibility of a reciprocal relationship, where academic emotions might also conversely affect self-efficacy. Testing this alternative reverse-mediation model falls outside the scope of the current cross-sectional analysis, but examining these reciprocal effects remains a valuable avenue for future longitudinal research.

## Figures and Tables

**Table 1:** Demographic Distribution of Survey Participants.

Category	Group	Number	Percentage
Gender	Male	615	58.74
	Female	432	41.26
Age	≤18 years	83	7.93
	19–21 years	406	38.78
	22–24 years	395	37.73
	≥25 years	163	15.57
Major	Arts	306	29.23
	Science	741	70.77
Student Type	Undergraduate	773	73.83
	Master’s	215	20.53
	Doctoral	59	5.64
Continent	Asia	540	51.58
	Europe	376	35.91
	Africa	131	12.51

**Table 2:** Descriptive Statistics and Correlation Analysis of Each Research Variable.

	1	2	3	4	5	6	7	8	9
1. Gender	—								
2. Age	−0.178	—							
3. Grade Level	0.305	−0.105	—						
4. Field of Study	0.022	0.123 **	−0.037	—					
5. Teacher Support	0.471 **	0.301 **	0.114 **	−0.564 **	—				
6. Self-Efficacy	0.271 **	0.207 **	0.104 **	0.344 **	0.402 **	—			
7. Academic Positive Emotions	0.227 **	0.201 **	0.067 **	0.348 **	0.467 **	0.027 **	—		
8. Academic Negative Emotions	−0.107 **	−0.074 **	−0.071 **	−0.025 **	−0.015 **	−0.028 **	−0.014 **	—	
9. Academic Anxiety	−0.127 **	−0.077 **	−0.081 **	−0.014 **	−0.019 **	−0.009 **	−0.014 **	0.141 **	—
*M*	3.117	3.094	2.767	3.815	3.845	3.514	2.214	2.109	2.014
*SD*	0.478	0.684	0.677	0.655	0.651	0.684	0.768	0.748	0.814

Note: ** *p* < 0.01; the same below.

**Table 3:** Mediation Effect Analysis.

	Proportion of Indirect Effect	95% Confidence Interval		*p*
		Lower Limit	Upper Limit	
Teacher Support → Positive Academic Emotions → Academic Anxiety	2.17%	−0.004	−0.002	<0.05
Teacher Support → Self-Efficacy → Positive Academic Emotions → Academic Anxiety	1.71%	−0.005	−0.002	<0.001
Teacher Support → Self-Efficacy → Academic Anxiety	47.85%	−0.037	−0.041	<0.05
Teacher Support → Negative Academic Emotions → Academic Anxiety	19.01%	−0.022	−0.019	<0.05
Teacher Support → Self-Efficacy → Negative Academic Emotions → Academic Anxiety	29.26%	−0.034	−0.031	<0.05

## Data Availability

The data presented in this study are available upon request from the corresponding author.
